# Topical PDT in the Treatment of Benign Skin Diseases: Principles and New Applications

**DOI:** 10.3390/ijms161023259

**Published:** 2015-09-25

**Authors:** Miri Kim, Haw Young Jung, Hyun Jeong Park

**Affiliations:** Department of Dermatology, Yeouido St. Mary’s Hospital, The Catholic University of Korea, Seoul 150-713, Korea; E-Mails: gimmil@hanmail.net (K.M.); myparable@naver.com (H.Y.J.)

**Keywords:** photodynamic therapy, topical photosensitizer, benign skin disease, acne vulgaris, wart, photorejuvenation

## Abstract

Photodynamic therapy (PDT) uses a photosensitizer, light energy, and molecular oxygen to cause cell damage. Cells exposed to the photosensitizer are susceptible to destruction upon light absorption because excitation of the photosensitizing agents leads to the production of reactive oxygen species and, subsequently, direct cytotoxicity. Using the intrinsic cellular heme biosynthetic pathway, topical PDT selectively targets abnormal cells, while preserving normal surrounding tissues. This selective cytotoxic effect is the basis for the use of PDT in antitumor treatment. Clinically, PDT is a widely used therapeutic regimen for oncologic skin conditions such as actinic keratosis, squamous cell carcinoma *in situ*, and basal cell carcinoma. PDT has been shown, under certain circumstances, to stimulate the immune system and produce antibacterial, and/or regenerative effects while protecting cell viability. Thus, it may be useful for treating benign skin conditions. An increasing number of studies support the idea that PDT may be effective for treating acne vulgaris and several other inflammatory/infective skin diseases, including psoriasis, rosacea, viral warts, and aging-related changes. This review provides an overview of the clinical investigations of PDT and discusses each of the essential aspects of the sequence: its mechanism of action, common photosensitizers, light sources, and clinical applications in dermatology. Of the numerous clinical trials of PDT in dermatology, this review focuses on those studies that have reported remarkable therapeutic benefits following topical PDT for benign skin conditions such as acne vulgaris, viral warts, and photorejuvenation without causing severe side effects.

## 1. Introduction

The principle of photodynamic action was first described by Oscar Raab in 1890 when he noted the toxic effects of acridine orange, which showed activity as a photosensitizer when combined with light and oxygen by destroying *Paramecium caudatum* cells without apparent damage to the protozoa when used alone [1]. Von Tappeiner discovered in 1903 that the administration of eosin following irradiation with light led to oxygen-dependent tissue reactions and improvements in skin diseases such as condylomata lata, lupus vulgaris, psoriasis, syphilis, and skin cancers [2]. He termed this activity a “photodynamic reaction”. In the late 1970s, photodynamic therapy (PDT) with hematoporphyrin derivative (HPD) was developed by Thomas Dougherty and his co-workers. They purified HPD to some extent and discovered that the administration of HPD followed by irradiation with red light resulted in oxygen-dependent tissue reactions [3].

Today, it is known that PDT relies on the absorption of harmless visible light by a photosensitizer, which then produces reactive oxygen species (ROS) such as singlet oxygen that destroy cancer cells, blood vessels, and pathogenic microorganisms. Currently, topical PDT is being widely used to treat actinic keratosis, and it has been studied for the treatment and prevention of superficial skin cancers in immunosuppressed patients for over 40 years. Additionally, the range of off-label indications has been expanding continuously.

Topical photosensitizers are used mainly in the field of dermatology because they can be delivered directly to the skin and rarely cause prolonged phototoxicity, a known side effect of systemic photosensitizers. Topical PDT is a new and rapidly evolving therapeutic option for the treatment of inflammatory skin diseases, such as psoriasis, acne vulgaris, and sarcoidosis, as well as infectious skin diseases, including verruca vulgaris, condyloma acuminatum, and cutaneous leishmaniasis [4,5,6,7,8,9].

Recent publications have reviewed the roles of PDT in precancerous skin lesions and superficial skin cancers [10,11]. In this review, we summarize the underlying principles in the use of PDT for dermatological conditions and discuss the clinical evidence for the use of topical PDT in treating acne vulgaris, photodamaged skin, and human papillomavirus infections, such as warts.

## 2. Mechanism of Action of Photodynamic Therapy (PDT)

Generally, PDT requires three essential components for the biochemical process to proceed: a photosensitizer, an appropriate light source, and tissue oxygen. The photosensitizer is a photosensitive molecule that is localized within the target tissue and is activated by a specific wavelength or energy of light. When the photosensitizer is exposed to light of the appropriate wavelength, it is activated from the ground state, S0, to the first excited state, S1.

The excited photosensitizer goes from S0 to S1 and undergoes intersystem crossing to the long-lived metastable triplet state that then transfers hydrogen or energy to ground state O_2_. This activated photosensitizer can then undergo two types of reaction in which the energy released can mediate selective cell killing. First, it can react directly with a substrate, such as intracellular molecules or cell membrane components, to form radicals, which then interact with oxygen to generate ROS (type I reaction); Second, the activated photosensitizer can transfer its energy directly to oxygen to form the ROS singlet oxygen (^1^O_2_), which further oxidizes various substrates (type II reaction). These species can oxidize various substrates and initiate cytotoxic effects by inducing necrosis and apoptosis. The generation of singlet oxygen species by type II photochemical reactions is believed to be the predominant reaction in PDT. At low levels of PDT, biological systems may be positively stimulated by low enhancement of ROS levels.

With blue light, activation of the Soret band causes the photosensitizer to go to S2 (the second electronically excited state). It then undergoes internal conversion to S1, after which one may either get fluorescence, further internal conversion to S0, or intersystem crossing to give the metastable triplet state which can either react with surrounding molecules (by H atom transfer) or transfer its excitation energy to ground (triplet) state oxygen to make single oxygen.

Beyond direct phototoxic effects on target tissues, PDT can stimulate diverse immune cells and inflammatory cell mediators, whereas the inverse was stated above. Immune-specific responses during PDT include the production of various cytokines, such as interleukin (IL)-1-β, IL-2, and tumor necrosis factor-α; matrix metalloproteinase (MMP)-1 and MMP-3 are also secreted by fibroblasts in response to PDT [12,13]. Following a low light dose, PDT with various photosensitizers has been shown to modify cytokine expression and induce immune-specific responses, resulting in immunomodulatory effects in inflammatory skin disorders.

## 3. PDT Components

### 3.1. Photosensitizer

In the mid-1900s, most photosensitizers used in PDT were derivatives of hematoporphyrin, an endogenous porphyrin that is formed in the first step of the heme biosynthetic pathway. Because large amounts of hematoporphyrin are required for photosensitization, the endogenous porphyrin can cause severe phototoxicity and prolonged and pronounced photosensitivity. Kennedy *et al.* developed a topical photosensitizer precursor, 5-aminolevulinic acid (ALA), in 1990, and this overcame many of the limitations of earlier PDT [14]. Currently, the most commonly used precursors of protoporphyrin IX (PPIX) in dermatology are topical 5-ALA, methyl-ALA (MAL), and other intermediate photosensitizing porphyrins. After topical application of the photosensitizer precursor, an “occlusion time” is permitted for the drug to be metabolized and for porphyrins to accumulate before activation with visible light. The advantages of topical PDT are the ability to treat multiple lesions simultaneously, low invasiveness, good tolerance, and excellent cosmetic results.

Given the ease with which photosensitizers and light can be delivered to the skin, it is not surprising that PDT is increasingly used as a therapy in dermatology. PDT is now used widely for the treatment of various skin tumors and infectious or inflammatory skin disorders.

#### 3.1.1. 5-Aminolevulinic Acid (ALA)-Induced Protoporphyrin IX (PPIX)

The application of ALA in PDT for skin disorders was first introduced in 1990 [14]. The main advantage of topical ALA-PDT is the absence of systemic cutaneous photosensitivity. The molecule 5-ALA is an amino acid and a precursor of PPIX. Topically applied ALA enters into cells of the epidermis and its appendages and is converted endogenously via the porphyrin pathway into PPIX, the active photosensitizing compound [14,15]. PPIX selectively accumulates in malignant cells, as well as in epidermal cells, sebaceous glands, and hair follicles [16].

Kennedy *et al.* observed that ALA applied topically in aqueous solution passed readily through abnormal keratin and was taken up more by altered keratinocytes than by normal keratinocytes [14]. Moreover, porphyrin biosynthesis is increased in malignant or premalignant cells, which means that PPIX photosensitization can be selectively induced in the abnormal epithelium [17]. After irradiating light within the action spectrum (including 400–410 and 635 nm), PPIX is activated and generates singlet oxygen or ROS, which cause selective cellular damage including damage to the plasma membrane, mitochondria, and endoplasmic reticulum [18].

ALA is available commercially as Levulan^®^ (DUSA Pharmaceuticals, Wilmington, MA, USA), which is a 20% 5-ALA solution in alcohol and is currently approved in combination with blue light for the treatment of nonhyperkeratotic actinic keratosis of the face and scalp in North America [18]. In Europe, ALA is available in the form of a patch containing ALA and a gel formulation of ALA in a nanoemulsion. It is also licensed for the treatment of actinic keratosis in combination with red light. The fact that ALA is a water-soluble amino acid, has low lipid solubility, and is unable to penetrate through the stratum corneum restricts its clinical application in PDT to superficial skin diseases such as actinic keratosis, Bowen’s disease, and superficial basal cell carcinoma (BCC) [19,20].

To enhance ALA delivery to target tissues, novel preparations of ALA, particularly nanoparticulate delivery vehicles, have been developed [21]. These nanoemulsion formulations can increase liposomal penetration and more selectively transport ALA to target tissues. ALA-PDT using nanoemulsion formulations has been recently shown to be superior to MAL-PDT in the treatment of actinic keratosis, with complete clearance rates of 78.2% and 64.2%, respectively [22].

#### 3.1.2. ALA-Ester-Induced PPIX

As a doubly charged molecule, ALA does not easily pass through cell membranes. Esterified derivatives of ALA that increase the lipophilicity of 5-ALA, such as MAL, butyl-ALA and hexyl-ALA, could potentially enhance penetration of the cell membrane and lead to more homogeneous tissue distribution of PPIX [23]. Once they have penetrated in the tissue, the ALA esters will be cleaved (hydrolysed) by the esterases present in the tissue and thus returned to active 5-ALA.

A methyl ester of ALA, MAL is a more recently introduced topical photosensitizer precursor used in the treatment of nonmelanoma skin cancer, including BCC and squamous cell cancer. When applied topically, MAL is metabolized into a photoactive porphyrin, PPIX, by a mechanism similar to that of ALA.

MAL is available in a cream containing 168 mg/g of MAL (final MAL concentration of 16.8%) and is marketed as Metvix^®^ (Galderma, Fort Worth, TX, USA) for the treatment of actinic keratosis and, depending on the country, for squamous cell carcinoma *in situ* and superficial and nodular BCC considered unsuitable for surgical procedures in Europe, Australia, and South America. In the United States, MAL is marketed as Metvix^®^ and is currently FDA approved for the treatment of nonhyperkeratotic actinic keratosis of the face and scalp in immunocompetent patients.

Many clinical trials studying the use of MAL, including phase III randomized controlled studies of MAL-PDT, support the utility of MAL-PDT in the treatment of malignant skin cancers [24,25,26,27]. Compared with ALA, MAL is a more lipid-soluble derivative because it is more lipophilic and therefore has a deeper skin penetration. However, there was no statistically significant difference in efficacy between ALA and MAL in the treatment of nodular BCC in one small pilot study or in the treatment of actinic keratosis in another randomized trial [28,29,30]. In theory, MAL may be more selective than ALA in its affinity for lipophilic environments, such as sebum, and thus would be expected to have greater efficacy in the treatment of acne [31].

#### 3.1.3. Other Topical Photosensitizers

Other photosensitizers that have been studied as alternatives to ALA and MAL include hypericin, chlorophyll, indocyanine green (ICG), and indole-3-acetic acid (IAA) [32,33,34]. Hypericin, a naturally occurring photosensitizer, is extracted from *Hypericum perforatum* plants. Hypericin-PDT has been shown to be effective in the treatment of dermatophytes, such as *Candida* spp. and *Trichophyton* spp. [35].

Chlorophyll has a structure similar to protoporphyrinogen IX and acts as a photosensitizer when it binds to magnesium [36]. The absorption spectrum of chlorophyll ranges from 400 to 700 nm and shows two absorbance peaks at 415 and 630–664 nm [37]. Compared with ALA or MAL, chlorophyll has the advantages of being cost-effective, with a relatively short incubation time, and the ability to act as a photosensitizer in a shorter time. Chlorophyll may play an important role as a convenient alternative treatment modality for patients with acne who are intolerant to conventional therapies. Kim *et al.* conducted a split-face study of acne in Asian patients in which they compared the use of PDT with intense pulsed light (IPL) after 30 min of 19% a,b-chlorophyll solution incubation and IPL alone [34]. Chlorophyll also has several practical advantages, including shorter incubation times and cheaper cost, while having equivalent efficacy. Chlorophyll-based PDT appears to be able to reduce sebum excretion.

Jang *et al.* performed a comparative split-face, single-blind, clinical trial of PDT with ICG and IAA for the treatment of acne vulgaris. Thirty-four patients with acne were treated with IAA with green light (520 nm) on half of the face and with ICG with near-infrared radiation (805 nm) on the other half five times at 1-week intervals [38]. There were significant reductions in acne lesions in both treatment groups compared with the baseline. They concluded that PDT with either ICG or IAA is effective in the treatment of inflammatory and noninflammatory acne.

### 3.2. Light Sources for Topical PDT

It is important to choose an appropriate light source by considering the optimal photosensitizer to be used in PDT. To achieve the most efficient therapeutic effect of PDT, the selection of appropriate light with a proper wavelength corresponding to the area of the maximal porphyrin-activation spectrum in tissues is important. PPIX following ALA or MAL application has a strong absorption peak at 405 nm in the Soret band area of the spectrum (~405–420 nm) along with several smaller Q bands; the last peak is at 635 nm.

Multiple light sources, including light-emitting diodes (LEDs), Argon ion pumped dye lasers, simple slide projector lamps, and other broadband light devices, such as IPLs and pulsed dye lasers (PDLs), are used in PDT.

The wavelength of blue light ranges from 410 to 420 nm with a peak wavelength of 417 nm, which corresponds to the area of maximum PPIX light absorption. In the dermatology field, ALA-PDT with blue light for the treatment of actinic keratosis is one of the most widely accepted applications. Blue light sources, including BLU-U^®^ (DUSA Pharmaceuticals, Wilmington, MA, USA) and ClearLight^®^ (Lumenis, Santa Clara, CA, USA) systems, are FDA-approved devices. These blue light devices have FDA approval for the treatment of mild-to-moderate inflammatory acne vulgaris [39].

Longer wavelengths of light, such as red light, are desirable for thicker lesions, such as in Bowen’s disease or BCC. Because red light does not excite PPIX as intensely as blue light, a higher fluence is needed, usually 75–100 J/cm^2^, depending on the bandwidth of the light source.

Several other light sources that correspond to the action spectrum of PPIX are used, including IPLs, PDLs (585 nm), and natural sunlight. IPL, which provides a range of wavelengths of light, and flash lamp-pumped PDLs are efficient in activating PPIX [40]. The benefits of these light sources over blue light are the time efficiency, the possibility of accumulating pulses, and the possible improvement in treating associated vascular and pigmented lesions within the broad treatment of the lesions.

**Figure 1:**
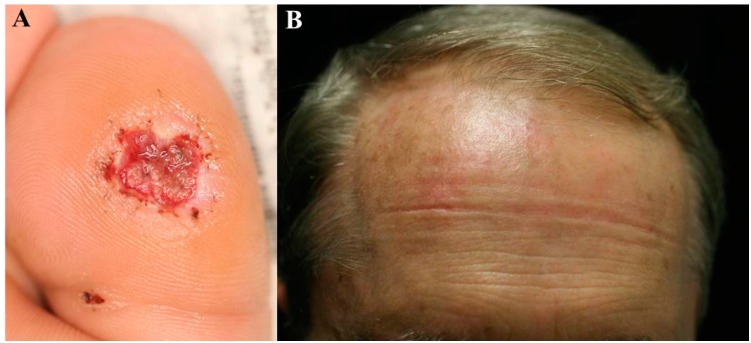
Side effects of PDT treatments. (**A**) Ulceration on the great toe after treatment of a wart with ILI-PDT; and (**B**) Diffuse mild erythema on the forehead after first treatment session of actinic keratosis with chlorophyll-PDT. ILI: intralesional injection; PDT: photodynamic therapy.

Side effects that may occur with PDT include erythema, swelling, ulceration, burning, or prickly sensation in PDT-treated sites [41] (Figure 1). Pain is most common side effect of topical PDT and may be often severe. This can be avoided nearly completely by lowering the fluence rate (mW/cm^2^) [42,43].

## 4. Therapeutic Applications of PDT in Benign Skin Diseases

### 4.1. PDT for Acne Vulgaris

Acne vulgaris is a chronic inflammatory skin disease that is characterized by excessive growth of bacteria such as *Propionibacterium acnes* in the sebaceous glands [44,45]. It commonly affects the face during adolescence and can lead to emotional, social, and psychological concerns such as anxiety, reduced self-esteem, and depression. Thus, the selection of the appropriate treatment option for each patient with acne vulgaris is important. The ideal treatment for acne vulgaris is an agent with antibacterial activity against *Propionibacterium acnes* and anti-inflammatory and seboregulating properties. Many treatment modalities are used to treat acne, including oral/topical antibiotics and retinoids, topical benzoyl peroxide, salicylic acid, azelaic acid, and various laser devices and surgical procedures [46,47]. However, the long-term use of oral/topical antibiotics may lead to resistance to antibiotics, the prevalence of which has been increasing [48]. Oral isotretinoin has greater potential risks such as intense dryness, systemic side effects, and birth defects [46].

PDT has been studied extensively in relation to acne vulgaris in recent years (Table 1). PDT has been shown to reduce sebum excretion and the amount of *Propionibacterium acnes*, and to improve the occlusion of the pilosebaceous orifices by promoting keratinocyte shedding [49]. PDT is a promising treatment modality for acne because it can inhibit sebum production and lead to prolonged remission of acne vulgaris without causing bacterial resistance to antibiotics. However, there is no consensus about the optimum light dosimetry and irradiance for the treatment of acne vulgaris. Given that *Propionibacterium acnes* produces large amounts of certain porphyrins, especially PPIX and coproporphyrin III, light sources alone with no added photosensitizer also have therapeutic photodynamic potential in the treatment of acne [50]. PDT using blue light has produced excellent clinical results in the reduction of inflammatory acne [51,52]. An open study of the effectiveness of phototherapy for acne vulgaris by Kawada *et al.* demonstrated that blue light phototherapy significantly decreased acne lesions by 64% in patients and reduced the number of bacteria *in vitro* [53]. In a double-blind randomized controlled trial, Kwon *et al.* treated 35 patients with mild-to-moderate acne with blue and red light-emitting diode devices [54]. Eighteen patients received 420-nm blue light and 660-nm red light for 2.5 min twice daily for 4 weeks as a single treatment; the other 17 patients, the control group, were treated with a sham device. During the final visit at 12 weeks, significant reductions of both inflammatory and noninflammatory acne lesions were observed, by 77% and 54%, respectively, in the light-treated group. Histopathology showed reductions in inflammatory cells and decreased sizes of sebaceous glands [54].

Hongcharu *et al.* reported the first clinical trial using ALA in the treatment of inflammatory acne vulgaris. In that study, 22 patients with inflammatory acne vulgaris had their face treated for an incubation time of 3 h followed by a 550–700-nm broadband light source. The number of inflammatory acne lesions and sebum secretion levels were significantly reduced after four treatment sessions at the 20-week follow-up [55]. Hongcharu *et al.* also reported histological evidence of the destruction of sebaceous glands, suggesting that one mechanism of action of PDT in acne treatment involves phototoxic effects on sebaceous follicles, inhibitions of sebaceous gland functions, and significant decreases in the number of bacteria.

**Table 1:** Clinical studies of topical PDT for the treatment of acne vulgaris.

First Author, Year [Reference]	Type of Acne and Location	Number of Patients	Photosensitizer (Contact Time)/Light Source	Number of Treatment Sessions (Follow-up Time)	Clinical Results
Hongcharu, 2000 [55]	Inflammatory, mild to moderate/back	22	20% ALA (3 h)/red light (550–570 nm) *vs*. red light only *vs*. placebo	Two randomized groups: 1 *vs.* 4 sessions (20 weeks)	ALA-PDT 1 session better than red light alone; After 20 weeks, 50% reduction of lesions after 4 sessions *vs.* ~30% reduction with 1 session
Itoh, 2001 [56]	Comedonal or inflammatory/face	13	20% ALA (4 h)/broad-spectrum (600–700 nm) halogen lamp	1 (24 weeks)	After 1 month, 100% some improvement without new lesions; at 3 months, 38.4% “excellent” response without new lesions
Goldman, 2003 [52]	Inflammatory, mild to moderate/face	22	20% ALA (15 min)/blue light (417 ± 5 nm) *vs.* blue light only	2 (2 weeks)	Reductions in inflammatory lesions: 68% with ALA-PDT *vs.* 40% with blue light only
Hong, 2005 [57]	Inflammatory, mild to moderate/face	8	20% ALA (4 h)/halogen lamp red (630 ± 63 nm)	1 (24 months)	Reductions in inflammatory lesions: 41.9% in treated sites *vs.* 15.4% in control
Wiegell, 2006 [58]	Inflammatory/Face	15	20% ALA *vs.* 16.8% MAL (3 h)/noncoherent red (630 nm)	1 (12 weeks)	Reductions in inflammatory lesions: 59% with both ALA and MAL; no significant difference between MAL and ALA sites
Rojanamatin, 2006 [59]	Inflammatory/face	14	20% ALA (30 min)/IPL (cutoff filter, 560–590 nm)	3 (12 weeks)	Reductions in inflammatory lesions: 87.7% for ALA-IPL *vs*. 66.8% for IPL only; not significantly different
Yeung, 2007 [60]	Inflammatory/face	23	16.8% MAL (30 min)/IPL (530–750 nm)	4 (12 weeks)	Reductions in inflammatory lesions: 65% with MAL-PDT *vs.* 23% with IPL only; noninflammatory lesions: 38% *vs.* 44%
Kim, 2009 [32]	Mild to moderate/face	16	0.06% ICG solution (30 min)/near-infrared diode laser (805 nm)	1 *vs.* 3 (8 weeks)	Subjective satisfaction score significantly higher in multiple-treatment group compared with a single-treatment group
Jang, 2011 [38]	Mild to moderate/face	34	IAA (30 min) with green light (520 nm) *vs.* ICG (15 min) with near-infrared radiation (805 nm)	5 (3 months)	Reductions in inflammatory and noninflammatory lesions and sebum secretion: significant reductions for both IAA and ICG; no significant differences between IAA and ICG
Kim, 2012 [34]	Mild to moderate/face	4	19% a,b-chlorophyll solution (30–60 min)/IPL (530–750 nm)	3 (4 weeks)	All subjects: mild improvement after three sessions; significant reduction in lesion count at 1-month follow-up
Kwon, 2013 [54]	Mild to moderate/face	55	None/home use, combination blue–red LED (660 and 420 nm) *vs.* control (sham device)	Twice daily for 4 weeks (12 weeks)	At 12 weeks, reductions in both inflammatory and noninflammatory acne lesions
Yin, 2014 [61]	Inflammatory, moderate to severe/face	40	15% ALA/ablative fractional Er:YAG laser + red light (633 ± 6 nm)/2 h	PDT: 4; Er:YAG laser: 5 (12 months)	After 6 months, 100% overall improvement in inflammatory lesions; 80% overall improvement in acne scars without recurrence

ALA, 5-aminolevulinic acid; MAL, methyl aminolevulinate hydrochloride; IPL, intense pulse light; LED, light-emitting diode; ICG, indocyanine green; IAA, indole-3-acetic acid; Er:YAG, erbium:yttrium aluminium garnet.

Hongcharu *et al.* reported the first clinical trial using ALA in the treatment of inflammatory acne vulgaris. In that study, 22 patients with inflammatory acne vulgaris had their face treated for an incubation time of 3 h followed by a 550–700-nm broadband light source. The number of inflammatory acne lesions and sebum secretion levels were significantly reduced after four treatment sessions at the 20-week follow-up [55]. Hongcharu *et al.* also reported histological evidence of the destruction of sebaceous glands, suggesting that one mechanism of action of PDT in acne treatment involves phototoxic effects on sebaceous follicles, inhibitions of sebaceous gland functions, and significant decreases in the number of bacteria.

**Figure 2:**
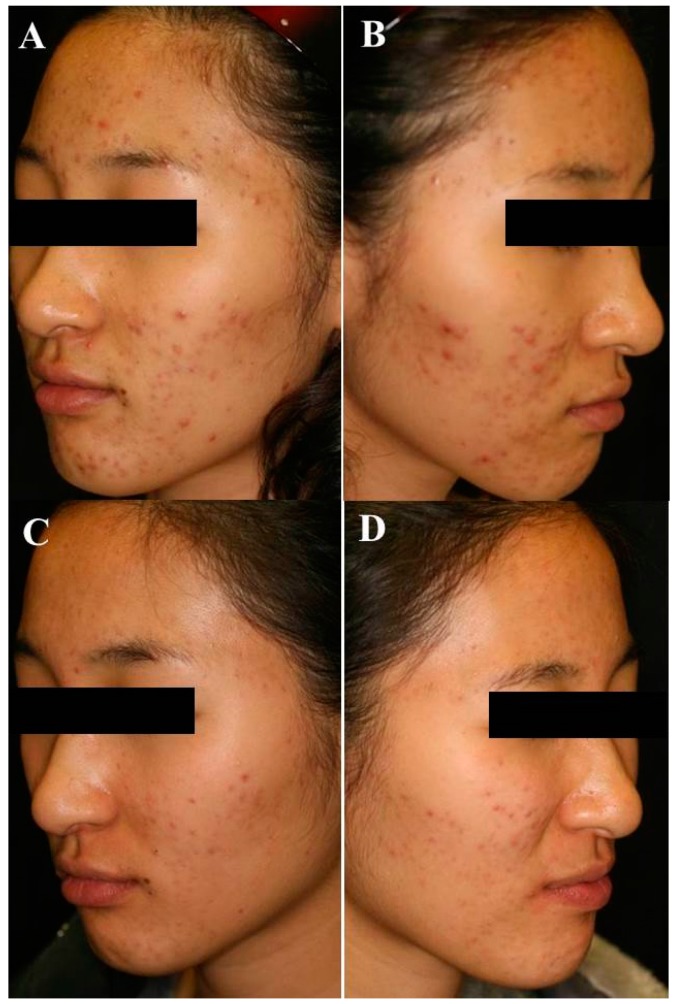
Representative photographs before (**A**,**B**) and after chlorophyll-PDT treatment (**C**,**D**). After three treatment sessions of chlorophyll-PDT, there was a significant decrease in the number of papules and pustules in moderate inflammatory acne patient [34].

In a case study by Itoh *et al.* using ALA with an incubation time of 4 h followed by a 635-nm pulsed excimer dye laser in a single patient with intractable acne vulgaris, the treated side remained disease free over the 8-month follow-up period [49]. In a subsequent study, Itoh *et al.* used a 600–700-nm halogen light source with a fluence of 13 J/cm^2^ after an ALA incubation time of 4 h, to treat 13 patients with acne vulgaris. Significant improvement in facial appearance was achieved and new acne lesions were reduced at 1, 3, and 6 months following PDT treatment [56]. The clinical improvement was maintained for at least 6 months. Kim *et al.* reported that Asian patients with acne felt a significant reduction in sebum secretion on the chlorophyll IPL-PDT-treated side compared with the IPL only-treated side [34] (Figure 2).

In a review of PDT studies for acne vulgaris using different light sources with various incubation times, Sakamoto *et al.* concluded that long-term remission was associated with an incubation time of at least 3 h, that ALA-PDT and MAL-PDT with high fluence and red light had similar efficacy, and that red light PDT may be more likely to inhibit and destroy sebaceous glands, resulting in higher response rates [62].

Moreover, PDT has been shown to be effective in reducing scar formation, and repeated treatment can improve scars, stimulating the wound-healing response. Yin *et al.* recently conducted a prospective pilot clinical trial to evaluate the efficacy of a combination of ALA-PDT and an ablative fractional erbium:yttrium aluminium garnet (Er:YAG) laser (2940 nm) [61]. Forty patients with severe acne were treated with 15% ALA-PDT four times, and then received ablative fractional Er:YAG laser treatment five times at one-month intervals. After 12 months, most of the patients had significantly decreased hypertrophic/atrophic scars without recurrence of inflammatory lesions. These findings suggest that ALA-PDT, in combination with ablative fractional Er:YAG laser treatment, may be a successful alternative for controlling the inflammation and decreasing scar formation in the treatment of severe acne.

The first reported clinical trial using MAL was reported by Wiegell and Wulf. They performed a randomized, comparative, investigator-blinded, split-face trial in 21 patients with acne [58]. They compared MAL-PDT with ALA-PDT and found no statistically significant difference in improvements in inflammatory or noninflammatory acne lesion counts at the 12-week follow-up. ALA-PDT caused more prolonged adverse effects such as erythema, edema, and scar formation after treatment. By contrast, Wiegell *et al.* reported that sites treated with MAL-PDT were less painful than were those treated with ALA-PDT in normal skin [63].

### 4.2. PDT for Refractory Palmoplantar Warts

Human papillomaviruses can cause various diseases, including warts, cervical carcinoma, anogenital squamous cell carcinoma, and papillomatosis. Among these conditions, warts are the most common entity caused by this type of virus. Most human papillomaviruses cause specific types of warts in certain anatomical locations, such as plantar warts, common warts, and genital warts. Management of warts is based on their clinical appearance and location, and on the immune status of the patient. The treatment options include surgical excision, cryotherapy, curettage, intralesional bleomycin, CO_2_ laser therapy, topical cytotoxic medications (5-fluorouracil, dinitrochlorobenzene), infrared coagulation, PDL, PDT, and electrosurgery. However, some lesions remain recalcitrant to therapy, and many recur after successful treatment [64].

Several studies have shown that ALA-PDT can be used to successfully treat cutaneous warts without causing significant side effects and with satisfactory cosmetic results. ALA-PDT with white light (halogen lamp; 250 W Osram; delivered via slide projector) was found to be more efficacious than red or blue light and standard cryotherapy [65]. One case report described the use of fractional resurfacing to aid PDL-PDT delivery to a recalcitrant plantar wart [66]. Another case report suggested that intralesional administration of ALA with a short incubation period was a safe and effective treatment for recalcitrant warts [67]. One advantage of PDT is the ability to treat a large surface area with minimal scarring.

**Figure 3:**
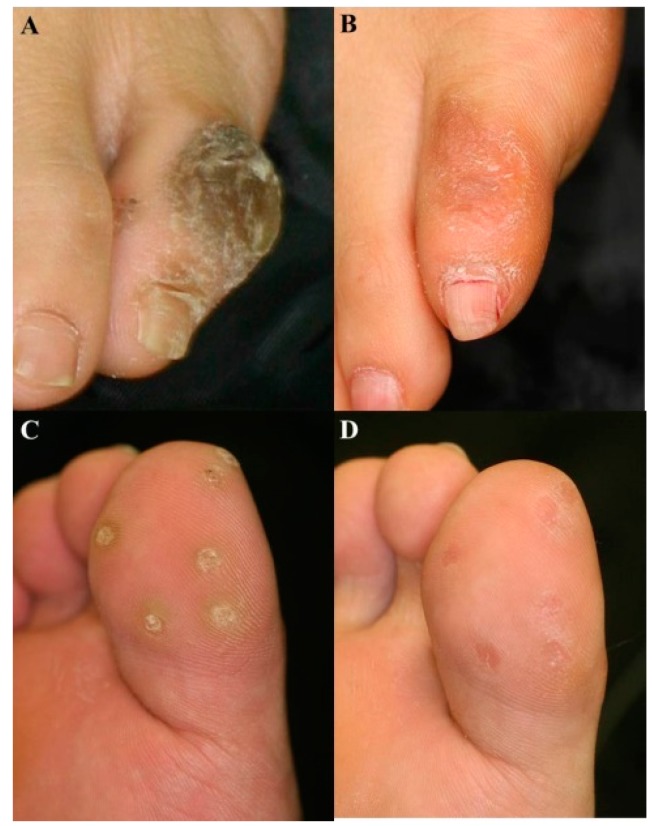
Representative photographs before and after ILI-PDT. Wart lesions on the foot at baseline (**A**,**C**) and 1 month after three sessions of ILI-PDT (**B**,**D**). Marked reduction of warts was shown on the great toe and little toe [67].

In a trial reported by Kim *et al.*, intralesional injection (ILI) combined with PDT was used to increase the efficacy of PDT in the treatment of viral warts. Eight patients with multiple viral warts on their hands and feet were treated with IPL after an injection of ALA solution directly into the warts. The wart clearance rates were about 60% after this treatment (Figure 3). The authors proposed that ILI-PDT may be a new therapeutic strategy for the treatment of thick recalcitrant viral warts [67].

However, few practitioners routinely use PDT for viral warts, possibly because of the lack of a standardized protocol. Clearance rates of recalcitrant hand and foot warts vary between studies, although rates of 56%–100% have been reported. In a randomized trial, six repeated ALA-PDT treatments showed superior results to those of a placebo group; the median reduction in wart area was 98% with PDT and 52% with placebo, although PDT induced intense pain in some patients [68]. PDT has been shown to achieve superior clearance compared with cryotherapy in a randomized pilot study of ALA-PDT in 30 patients with recalcitrant warts [65].

The success of ALA-PDT in treating a patient with multiple facial plane warts has been reported, and a recent case series using a 10% ALA formulation was reported to clear facial warts after two sessions in 17 of 18 patients with only one recurrence after 6 months [69,70]. Complete clearance of periungual hand warts was achieved in 18 of 20 patients (36/40 warts) using ALA-PDT after a mean of 4.5 fortnightly treatments [71]. MAL-PDT was effective in treating a recalcitrant hand wart in a case report, although further studies are needed on its use in warts generally [72].

### 4.3. PDT for Genital Warts

Topical PDT is a treatment option for patients with genital warts. The use of PDT in conjunction with 5-ALA, Photolon^®^ (Belmedpreparaty, Minsk, Republic of Belarus), polyhematoporphyrin, Er:YAG laser, optical parametric oscillator (OPO) laser irradiation, or CO_2_ laser vaporization has been suggested [73,74,75,76,77,78,79]. In a large study of 164 patients with urethral condylomata, ALA-PDT cleared 95% of lesions, and only 5% recurred after 6–24 months [80]. A randomized study compared a single treatment of ALA-PDT with a conventional CO_2_ laser treatment in 65 patients with condyloma acuminata. Clearance rates were 95% with ALA-PDT and 100% with the conventional CO_2_ laser, and the persisting lesions cleared after repeated PDT treatments [73].

In another large randomized clinical trial of ALA-PDT involving 90 patients with condylomata acuminata, all lesions were cleared with both treatment modalities (PDT *vs.* CO_2_ laser), although there were fewer recurrences after PDT at 3 months (9% *vs.* 17%, respectively) [81]. However, ALA-PDT was not shown to be beneficial as an adjunctive treatment to ablation of condyloma acuminata with a CO_2_ laser. In a large prospective randomized trial involving 175 patients, the cumulative recurrence rate of the lesions ablated with the CO_2_ laser in conjunction with ALA-PDT was 50% *vs.* 53% with CO_2_ laser vaporization alone at the 12-week follow-up [82].

### 4.4. PDT for Photorejuvenation

Photoaging of the skin is a complex, progressive biological process. Clinically, photoaging is characterized by wrinkles, dryness, roughness, laxity, and irregular pigment changes. Recently, PDT delivered with various lasers and light sources, such as IPL, PDL, and blue light-emitting lamps, has been shown to improve photodamaged skin and its associated actinic keratosis and to have excellent cosmetic results [83,84]. With the development of short-contact ALA-PDT and synergy between the photochemical effects (ROS) and the photothermal effects (using IPL or PDL), the application of PDT could be extended to the cosmetic field [84].

In a randomized, controlled, split-face study of 25 patients with sun-damaged skin, MAL-PDT followed by irradiation with either an LED (635 nm, 37 J/cm^2^) or an IPL showed significant improvement in wrinkling and mottled pigmentation at 3 months [85]. In a split-face study, IPL-assisted ALA-PDT was compared with IPL alone in 16 patients in a side-by-side setup. IPL-assisted ALA-PDT achieved greater improvements in photodamaged skin and greater clearance of actinic keratosis lesions than did IPL alone. The authors suggested that ALA-PDT may have useful applications for photorejuvenation in the future [86].

Recently, Shin *et al.* evaluated IPL-PDT and a long-pulsed neodymium-doped yttrium aluminium garnet (Nd:YAG) laser (LPNY) using a 0.5% ALA liposomal spray for periorbital wrinkles in Asian patients. The patients were exposed to three PDT treatments every 3 weeks and the investigators followed up 3 months after the last treatment. The investigators found greater wrinkle reduction on the IPL-PDT-treated side than on the LPNY-treated side [87]. These results are supported by several studies that showed increases in both collagen production and epidermal proliferation [88]. Marmur *et al.* reported that PDT using IPL promoted a greater amount of type I collagen than did IPL treatment alone and suggested that PDT is superior to other nonablative laser therapies [89]. Consistent with these other studies, Orringer *et al.* also found that collagen production and epidermal thickness were increased after ALA-PDT using a PDL [90].

## 5. Conclusions

The use of PDT for the treatment of premalignant and malignant skin lesions, such as actinic keratosis, BCC, and Bowen’s disease, is established. The introduction of topical photosensitizers, which are convenient and less phototoxic, has expanded the range of clinical application of PDT in dermatology. There is growing evidence that topical PDT is effective in the treatment of various benign skin conditions including viral warts, photodamaged skin, and acne vulgaris, and especially in lesions that are inflamed or oily and unresponsive to conventional therapies. With increasing concern about bacterial resistance due to the long-term use of oral and topical antibiotics for acne vulgaris, topical PDT is an effective method for treating acne vulgaris because it inhibits sebaceous gland function and reduces the number of bacteria, resulting in prolonged remission of acne vulgaris. In addition, topical PDT effectively improves photodamaged skin, including skin laxity and mottled pigmentation. However, the use of PDT in dermatology has not been optimized. More well-controlled clinical studies of various skin conditions and diseases in a large number of patients with long-term follow-up are needed to standardize the type of light source, wavelength, treatment parameters, and photosensitizer incubation time.

Future advances hold great promise for the treatment of various skin diseases and the combination of PDT with other treatments. Such advances include the targeting of photosensitizers using molecular recognition and incorporation of nanotechnology (*i.e.*, photosensitizer conjugated to nanoparticles); development of further optimizing the ALA derivatives, *i.e.*, not only considering methyl-ALA but also hexyl-ALA or even ALA-DGME; light delivery methods, and dosimetry techniques; use of antimicrobial photoinactivation; and the influence of host immunity.
